# Fear and Uncertainty Do Not Influence Reported Willingness to Undergo Lumbar Punctures in a U.S. Multi-Cultural Cohort

**DOI:** 10.3389/fnagi.2017.00022

**Published:** 2017-02-10

**Authors:** Dobromira Z. Tsvetkova, Sharon H. Bergquist, Monica W. Parker, Thomas L. Jarrett, Jennifer C. Howell, Kelly D. Watts, Alexander Kollhoff, David L. Roberts, William T. Hu

**Affiliations:** ^1^Department of Neurology, Emory University School of Medicine, AtlantaGA, USA; ^2^Center for Neurodegenerative Diseases Research, Emory University School of Medicine, AtlantaGA, USA; ^3^Division of General Internal Medicine and Geriatrics, Department of Medicine, Emory University School of Medicine, AtlantaGA, USA; ^4^Alzheimer’s Disease Research Center, Emory University School of Medicine, AtlantaGA, USA

**Keywords:** Alzheimer’s disease, cerebrospinal fluid, health services research

## Abstract

Cerebrospinal fluid (CSF) biomarkers for Alzheimer’s disease and related disorders can provide early and accurate prediction of underlying neuropathology even when the clinical symptoms are mild, but lumbar punctures (LP) to obtain CSF can be perceived as frightening and invasive. We previously demonstrated that this negative perception of the LP is strongly associated with a negative LP experience in terms of discomfort and complications, but it is not known what factors can lead to a negative perception of the LP. It has also been proposed that LP is less well-perceived by adults in the U.S. compared to Europe and elsewhere, although there is a paucity of primary data to support this. To address these knowledge gaps, we conducted a survey of 237 younger and older adults in the Atlanta area including a significant number born outside of the U.S. (*n* = 82, 34%) to determine demographic, medical, and experiential factors associated with the perception of LP as well as the willingness to undergo LP for medical or research purposes. Our results show that one in four respondents in this cohort with limited first-hand LP experience viewed the LP as a frightening invasive procedure, but the majority (89%) were willing to undergo LP for medical reasons. General awareness of the LP was associated with both standard and negative views of the LP, but perception did not influence willingness to undergo the procedure. Multi-variate models showed that higher annual household income, not place of birth or past experience, was associated with greater willingness to undergo LPs. We conclude that Americans (born in the U.S. or abroad) are not resistant to LPs if there is useful information to improve their health, although there is limited enthusiasm to undergo LPs solely for research purposes. At the same time, we failed to find modifiable factors to improve the perception of LP among those who already perceive it as frightening and invasive. Future recruitment effort should target adults with no preconceived notion of the LP with emphasis on data related to safety and tolerability.

## Introduction

The lumbar puncture (LP) is a well-known procedure to obtain cerebrospinal fluid (CSF) for the detection of infection, inflammation, and malignancy involving the central and peripheral nervous system. (Powers, 1987; Straus et al., 2006; Majed et al., 2009; Doherty and Forbes, 2014) Following the discovery that the main pathogenic proteins in Alzheimer’s disease (AD) and Parkinson’s disease are reflected in the CSF, (Vandermeeren et al., 1993; Motter et al., 1995) there have been renewed interests in developing CSF biomarkers for the diagnosis and prognosis of various neurodegenerative disorders. (Zhang et al., 2008; Hu et al., 2010, 2013; Craig-Schapiro et al., 2011) Among candidate biomarkers for neurodegenerative disorders in which direct brain biopsy is not practical, CSF biomarkers provide etiologic information at earlier stages with greater specificity than MRI, and currently provide higher overall accuracy at lower costs than cerebral PET imaging. (Mayeux and Stern, 2012; Weiner et al., 2013; Mehrabian et al., 2015) CSF testing is also more cost effective if more than one neurological etiology is considered (“one poke vs. four scans”), and can be preferred when patients have extreme claustrophobia or in areas where molecular imaging is not feasible. At the same time, well known obstacles exist in popularizing LPs as the main tool in early diagnosis of AD and related disorders. Aside from technical (anti-coagulation, degenerative spine disease) and clinician (lack of practice, need for radiology support) factors, older adults’ fear of the LP as an invasive procedure in North America (Menendez-Gonzalez, 2014) is often considered a major impetus for developing blood-based biomarkers or molecular imaging. (Grzybowski et al., 2011) However, there are very limited primary data to substantiate this fear, (Alcolea et al., 2014; Thakur et al., 2015; Magin et al., 2016), despite 400,000 LPs documented yearly in the US. (Evans et al., 2000) There is thus a need to analyze the prevalence of negative LP perception and factors which promote this fear.

We recently found negative pre-LP perception to predict greater discomfort and complications in an AD biomarker study. (Howell et al., 2016) In this cohort recruited from community AD seminars and existing AD research programs, only 6% of the study participants saw the LP as frightening and invasive and 15% were reluctant to undergo the procedure prior to the LP. This positive or neutral perception in a specialized cohort from a tertiary referral center may reflect altruism among research participants, and may not readily translate to the broader older population. As a follow-up study on older adults’ willingness to undergo diagnostic LPs, we surveyed a group of community-dwelling adults not previously exposed to AD community events or research to determine their perception of the LP as well as factors which influence it. Because clinicians in European and North American research consortiums and memory clinics have different opinions on the acceptability of LP among older adults, (Menendez-Gonzalez, 2014; Duits et al., 2016) we specifically surveyed respondents’ country of birth to see if older adults born in North America are more likely to view LP as frightening and invasive than those born elsewhere. Finally, we sought to determine if the perception of LP directly predicts the willingness to undergo LPs, as other factors (clinical benefits, future planning, and altruism) may confound the influence of perception.

## Materials and Methods

### Standard Protocol Approvals, Registrations, and Patient Consents

Because no protected health or other identifying information (other than categories) was collected, the Emory Institutional Review Board reviewed the study and determined the work to not require formal approval.

### Study Participants

Two hundred and thirty-seven participants were recruited from two sources in the greater metropolitan area of Atlanta during the period between February and May 2016. The participants were adults who attended community cultural events (*n* = 86) not related to aging, memory, or neurological diseases; and patients attending the Emory Primary Care Internal Medicine Clinic for routine preventative and illness related visits (*n* = 151). Compared to the Clinic cohort, respondents from community cultural events were more likely to be born abroad (76% vs. 11%, *p* < 0.001) and have lower annual household incomes (19% vs. 66% with greater than $60,000 per year), but were otherwise similar in age, gender, education, race, and self-reported health.

The survey consisted of four parts (Supplementary Data Sheet 1): (1) age range (<55, 55–64, 65–74,75–84, and >84), gender, self-identified race (Caucasian, African American, Asian, other), ethnicity (Hispanic, non-Hispanic), country of birth, education (high school or less, associate degree, bachelor’s degree, master’s degree, or more), household income range (according to quintiles from US census), self-reported health status (excellent, good, fair, and poor); (2) five true/false questions on knowledge related to AD correlated with scores on the AD Knowledge Scale which consists of 30 true/false question; (3) general awareness and experience with LP, including three yes/no questions on whether the respondent knows what a LP is, has had an LP in the past (if so, purpose, location, and complications), and knows someone who has had a LP in the past; (4) specific knowledge and attitudes toward LP related to diseases, including which neuropsychiatric (AD, Parkinson’s disease, multiple sclerosis, meningitis, depression) and non-neuropsychiatric illnesses (skin and gastrointestinal disorders) can be diagnosed by LP; perception of LP; respondent’s willingness to consider an LP for different purposes (early diagnosis, tailoring therapies, predicting future disease risk, research on disease respondent has, research on disease respondent does not have).

To assess perception of LP, we modified a question from the AD Center LP Pre-Survey (with permission from John Morris, MD, Washington University^1^). We had administered the prior version to a cohort of older African Americans and Caucasians undergoing LP for an AD biomarker study, and respondents chose between “a standard medical procedure” and “a frightening invasive procedure.” (Howell et al., 2016) Many respondents expressed uncertainty about the procedure, and we thus included a third option (“unsure”) in the current survey.

A total LP knowledge score was derived by the number of correct answers for conditions which can or cannot be diagnosed by LP. An overall LP willingness score was derived by number of purposes for which the respondent is willing to undergo an LP (maximum of 5). Three categorical variables were also derived to assess willingness to undergo LP, including LP for any reason, LP for clinical purposes (early diagnosis, tailoring therapies, predicting future disease risk), and LP for research purposes (related to disease one does or does not have).

### Statistical Analyses

All statistical analyses were performed using SPSS 22 (IBM SPSS, Armonk, NY, USA). Basic demographics were compared using Chi-squared tests for categorical variables. Effects from country of origin (US or abroad) were analyzed using Chi-squared tests for categorical variables, and Student’s *T*-tests for continuous variables (brief AD knowledge score, LP knowledge scores, and LP willingness score), with *p* < 0.01 to correct for multiple comparisons. To derive a multi-variate model that predicts favorable LP perceptions, univariate analyses (Chi-squared tests and Student’s *T*-tests) were first used to identify factors associated perceiving LP as a standard medical procedure at the *p* < 0.10 level, followed by multi-variate logistic regression analysis to identify variables independently associated with each of the three view (standard medical procedure, unsure, frightening invasive; variables were entered into the model if *p* < 0.05 and removed from the model if *p* ≥ 0.10). Similarly, univariate analyses (threshold of *p* < 0.10) followed by multi-variate linear regression analysis were used to identify factors associated with respondents’ willingness to undergo LP (variables were entered into the model if *p* < 0.05 and removed from the model if *p* ≥ 0.10).

## Results

One hundred and fifty-seven (66%) respondents were born in the US (**Table 1**). Compared to respondents born abroad, more respondents born in the US were 55 years old or older (76% vs. 60%, *p* = 0.009), African Americans (17% vs. 2%, *p* = 0.001), and had higher household incomes (65% vs. 28% with income >$60,000, *p* < 0.001). There was also a trend that respondent born in the US were more likely to have had first- or second-hand experience with undergoing LPs than respondents born abroad (48% vs. 33%, *p* = 0.025), but the two groups were otherwise similar in gender, education, and self-reported health.

**Table 1:** Respondent demographics according to country of birth (U.S. or abroad).

	Born in the U.S. (*n* = 155)	Born abroad (*n* = 82)	*p*
**Male (%)**	72 (46%)	35 (43%)	0.579
**Age**			0.001
<55	37 (24%)	33 (40%)
55–64	42 (27%)	32 (39%)
65–74	48 (31%)	12 (15%)
75–84	21 (14%)	5 (6%)
85 or older	7 (4%)	0
**Race**			0.003
African American	26 (17%)	2 (2%)
Non-Hispanic Caucasian	126 (81%)	72 (88%)
Asian	0	2 (2%)
Hispanic	1 (1%)	1 (1%)
Other	2 (1%)	5 (6%)
**Education**			0.112
Less than high school	1 (1%)	17 (11%)	
High school	13 (8%)	61 (39%)	
Associate degree	63 (41%)	0	
Bachelor’s degree	18 (22%)	4 (5%)	
Masters/doctorate degree	24 (29%)	36 (44%)	
**Self-reported health**			0.156
Excellent	46 (30%)	88 (57%)	
Good	17 (11%)	4 (3%)	
Fair	24 (29%)	42 (51%)	
Poor	16 (20%)	0	
**Annual household income**			<0.001
<$20,000	14 (9%)	25 (30%)	
$20,000–38,000	14 (9%)	21 (26%)	
$38,001–60,000	33 (21%)	14 (17%)	
$60,001–100,000	29 (19%)	12 (15%)	
>$100,100	65 (42%)	10 (12%)	
**Had a previous LP**	18 (12%)	5 (6%)	0.172
**Knew someone else who had a previous LP**	65 (42%)	24 (29%)	0.051
**Perception of LP**			0.721
A standard medical procedure	63 (41%)	29 (35%)	
A frightening invasive procedure	37 (24%)	22 (27%)	
Not sure	55 (35%)	31 (38%)	

**FIGURE 1:**
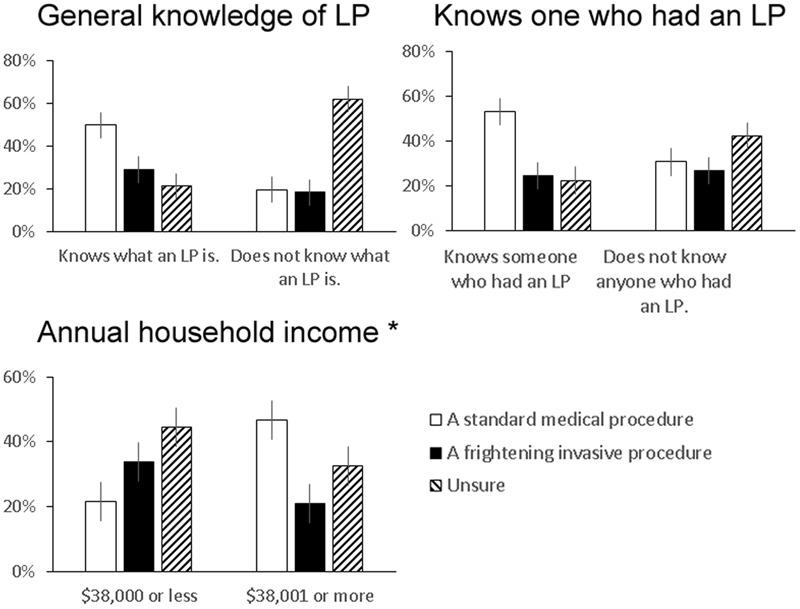
**Factors which influenced perception of lumbar puncture (LP) as a standard medical procedure or a frightening invasive procedure.** Univariate analyses showed that having basic knowledge of LP (*p* < 0.001), knowing someone (self or non-self) who has undergone an LP (*p* < 0.001), and higher income (*p* = 0.002) was each associated with the perception that LP was a standard medical procedure (proportion and 95% confidence intervals shown). ^∗^ Only income remained significantly associated with a neutral LP perception vs. a frightening invasive procedure in the multi-variate model. 95% confidence intervals are shown.

One in four respondents (59/237, or 25%) considered LPs as frightening or invasive, and this did not differ between respondents born in the US and abroad (24% vs. 27%, *p* = 0.72, **Figure 1**), between those who have or have not had themselves in the past (6/23 vs. 53/205, *p* = 0.82), and between those who knew or did not know someone else who had an LP in the past (21/89 vs. 38/139, *p* = 0.53). At the same time, those who had an LP in the past (61% vs. 38%, *p* = 0.034) or knew someone who had an LP (53% vs. 32%, *p* = 0.002) were more much more likely to view the LP as standard than those who did not. Therefore, first- or second-hand knowledge of LP was associated with a relative shift in the LP perception from “unsure” to “standard medical procedure,” but had minimal impact on the proportion who considered it as frightening and invasive.

**FIGURE 2:**
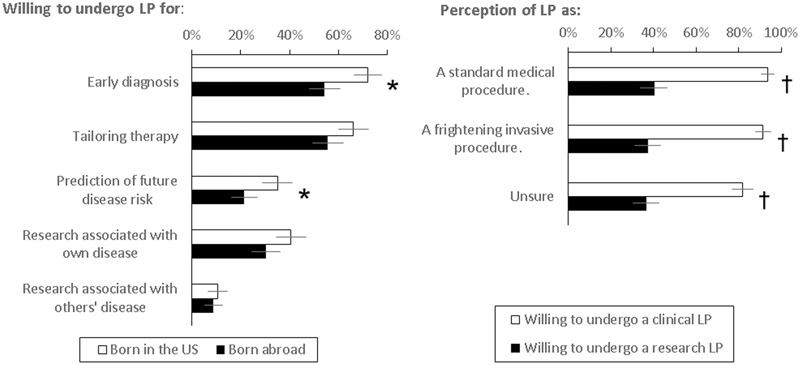
**Willingness of respondents born in the US or abroad to undergo LPs for different reasons (proportion and 95% confidence intervals shown).** ^∗^ Higher in respondents born in the US, *p* < 0.05; ^†^ greater willingness to undergo clinical than research LP, *p* < 0.001.

Univariate analysis showed general awareness of LP (*p* < 0.001), recruited from the Internal Medicine Clinic (*p* = 0.041), having at least a bachelor’s degree (*p* = 0.038), having good or excellent health (*p* = 0.018), annual household income >$38,000 (*p* < 0.001), and knowing someone (self or non-self) who has undergone an LP (*p* < 0.001) to each associate with the perception of LP as a standard procedure (**Figure 2**). Interestingly, knowledge about AD and LP did not differ according to pre-LP perceptions. Multi-nominal logistic regression analysis showed that higher income (3.347, 95% CI 1.542–7.264, *p* = 0.002) was associated with higher likelihood of viewing the LP as standard instead of frightening and invasive, and older age (2.717, 95% CI 1.236–5.988, *p* = 0.013) and not knowing anyone who has undergone an LP (2.070, 95% 0.977–4.383, *p* = 0.057) were associated with more uncertain than negative perception of LP (**Table 2**).

**Table 2:** Factors from multi-variate logistic regression analysis which influenced pre-procedure perception of LP as something other than a frightening invasive procedure.

	Factors	Exp (B) (95% C.I.)	*p*
Standard Medical Procedure	Annual household income >$38,000	3.347 (1.542, 7.264)	0.002
Unsure	Younger than 55 years of age	2.717 (1.236, 5.988)	0.013
	Had an LP or knew someone who had an LP	0.483 (0.228, 1.023)	0.057

Because one’s perception of an LP might influence his or her willingness to undergo an LP, we next analyzed the relationship between willingness to undergo an LP and demographic, knowledge, and experience factors. Univariate analyses showed that respondents born in the US were slightly more willing to undergo a LP for any clinical (92% vs. 83%, *p* = 0.044) and any research (43% vs. 30%, *p* = 0.072) purposes than respondents born abroad, especially for early detection (103/143 vs. 43/79, *p* = 0.008) and future disease risks (50/143 vs. 17/79, *p* = 0.037, **Figure 2**). Additional univariate analyses showed higher education (*p* = 0.006), higher income (*p* < 0.001), not being unsure (*p* = 0.019), and knowing someone (self or non-self) having undergone a previous LP (*p* = 0.025) to also associate with greater willingness. More knowledge about AD (*p* = 0.290) and the diagnostic role of CSF (*p* = 0.870) was not associated with willingness to undergo LPs, but being surveyed at the Primary Care Clinic was associated with significantly higher likelihood to undergo research LPs (52% vs. 16%, *p* < 0.001). Multi-variate linear regression analysis showed only higher education (coefficient = 0.211 for each higher category, 95% C.I. 0.027–0.395, *p* = 0.025) and higher income (coefficient = 0.256 for each higher category, 95% C.I., 0.125–0.386, *p* < 0.001), but not country of origin or perception of LP, to predict greater willingness to undergo an LP for any reason. Importantly, the majority of respondents were willing to undergo clinical LP for one of the reasons regardless of their perception of the procedure (**Figure 2**).

## Discussion

As a procedure, LPs have relatively few serious complications in experienced centers. (Peskind et al., 2005; Duits et al., 2016) We previously showed in a cohort of volunteers participating in a memory and aging study (including CSF collection) that the perception of LP as frightening and invasive was associated with a sixfold increase in self-reported complications. (Howell et al., 2016) Here we showed that general knowledge about the LP is associated with both neutral and negative perception of the LP, and the only factor that distinguished between the two groups with opposite LP perceptions was household income. We also found a dissociation between the perception of LP and the willingness to undergo the procedure, and neither was influenced by whether someone was born in the US. Thus, while most Americans expressed willingness to undergo LP for medical reasons, education aimed at those with limited awareness of LP – rather than those with formed opinions – is probably more effective in normalizing the public’s perception of the procedure.

Whereas our and other studies have examined the relationship between LP perception and experience, (Peskind et al., 2005; Alcolea et al., 2014; Duits et al., 2016; Howell et al., 2016) there is little information on the community’s perception of LP. Compared to our previous cohort already participating in research, the current cohort was much less likely to view the LP as a standard medical procedure (39% vs. 93%). The difference could be due to the introduction of a new response item (“not sure”) in the revised survey question, but there was also a greater proportion of respondents who viewed the LP as frightening and invasive compared to the prior cohort (25% vs. 7%). We did not quantitatively compare the two cohorts’ LP views because of the different response options, but it may not come as a surprise that the LP perception differs between research participants and the general population. In support of this difference, the community cohort is more willing to undergo LP for clinical reasons related to their own health than for research (89% vs. 38%). By comparison, our community cohort is less likely to undergo research LPs than previous cohorts at academic medical centers in Ann Arbor, MI, USA (84%; Kim et al., 2013) and Boston, MA, USA (64%), (Marcantonio et al., 2008) but differences in survey design makes direct comparison of these three cohorts challenging. Nevertheless, our findings urge caution in translating willingness to participate in research LPs to readiness to undergo clinical LPs.

Our original goal was to identify factors which reduce the likelihood of older adults perceiving the LP as frightening and invasive because of the association between this negative perception and post-procedure discomfort and complications. In our cohort, first- or second-hand experiences with LP did not reduce the proportion of people with negative LP perceptions, although this experience did reduce uncertainty about the procedure by shifting some adults’ opinions from “unsure” to neutral (standard operating procedure). Having had first- or second-hand experience also did not influence the willingness to undergo the procedure. One might have hypothesized those who had these exposures to have more negative attitude or lower willingness toward the procedure, as U.S. teaching institutions have observed a steady decline in the procedural comfort level among medical students and resident physicians. (Hicks et al., 2000; Wu et al., 2006; Mourad et al., 2010) On the other hand, advances including professional societies’ evidence-based practice parameters, (Lewis et al., 2002; Armon et al., 2005) regular use of radiological guidance, (Kroll et al., 2015) and a greater overall focus on patient safety, (Ormerod, 2014) may have compensated for the diminishing operator experience to stabilize the public’s perception of the LP. More importantly, the high proportion of respondents willing to undergo LP for clinical purposes contradicts the common belief that Americans are generally resistant to LPs, and at least some of the perceived challenges in conducting clinical or research CSF collection may reflect clinician bias, shifting models of care delivery, and low reimbursement rates for the LP than patient preference or safety (O’Connor and Lee, 1999; Kroll et al., 2015).

Because our group actively conducts CSF biomarker studies in specialized as well as community-based settings, (Hu et al., 2013, 2015) we are in a unique position to explore patient-centered issues related to the use of CSF biomarkers in AD research and diagnosis. While we identified higher household income to associate with a standard perception of LP, we do not know whether this relationship reflects greater familiarity and trust associated with the modern healthcare systems, availability of neuro-diagnostic tests, (McLane et al., 2015) and access to higher quality health information in families and neighborhoods with higher income. (Marmot, 2002; Borhani-Haghighi et al., 2009; Trujillo and Fleisher, 2013) Alternatively, those not familiar with LP may mistake it for an expensive procedure with limited insurance coverage. We did not survey factors associated with past contacts with healthcare systems, health insurance status, or trust in medical research (Williams et al., 2010) to identify mediators of the relationship. We also did not have information on respondents’ past exposure to neurological diseases (in family, friends, and media) which may strongly influence both the perception of the procedure and willingness to undergo it. However, we believe this study adds to the growing body of knowledge on the perception and acceptance of LPs, and sets the stage for prospectively testing their clinical utility, safety, and cost effectiveness beyond specialized research settings.

## Author Contributions

DT is responsible for conception/design of the study; acquisition, analysis, and interpretation of the data; and drafting of the manuscript. SB is responsible for acquisition and interpretation of the data; and critical revision of the manuscript for important intellectual content. MP is responsible for conception/design of the study; interpretation of the data; and critical revision of the manuscript for important intellectual content. TJ is responsible for acquisition and interpretation of the data; and critical revision of the manuscript for important intellectual content. JH is responsible for conception/design of the study; acquisition, analysis, and interpretation of the data; and drafting of the manuscript. KW is responsible for acquisition and analysis of the data; and critical revision of the manuscript for important intellectual content. AK is responsible for acquisition and analysis of the data; and critical revision of the manuscript for important intellectual content. DR is responsible for conception/design of the study; interpretation of the data; and critical revision of the manuscript for important intellectual content. WH is responsible for conception/design of the study; acquisition, analysis, and interpretation of the data; drafting of the manuscript. All authors have approved the submitted manuscript.

## Conflict of Interest Statement

The authors declare that the research was conducted in the absence of any commercial or financial relationships that could be construed as a potential conflict of interest.

## Footnotes

1 https://www.alz.org/research/funding/partnerships/global_biomarkers/US_LP_Survey.pdf
