# Supplementary material for: Fear and Uncertainty Do Not Influence Reported Willingness to Undergo Lumbar Punctures in a U.S. Multi-Cultural Cohort
Source: Front Aging Neurosci. 2017 Feb 10;9:22. doi: 10.3389/fnagi.2017.00022 (PMC5300987; doi:10.3389/fnagi.2017.00022)
Supplement: Supplementary file 1 [file Data_Sheet_1.pdf]

Knowledge, attitudes and experience towards Lumbar puncture

We are conducting a survey on the knowledge, attitudes and past experience related to having a lumbar puncture as part of a clinical test or a research study for brain diseases like Alzheimer's. Participating in this survey does not require you to undergo an actual medical procedure. I will be asking you a number of questions related to your background, your exposure to the medical system, and your experience with medical research.

First, I would like to know more about you. Please circle or check the information that best describes you.

1. Gender (circle one) Female Male
2. Age (circle one) < 55 55-64 65-74 75-84 > 84
3. Race ☐ African American ☐ Caucasian ☐ Asian ☐ Hispanic ☐ Other
4. Education: ☐Doctorate/Master's ☐Bachelor's ☐ Associates ☐ High school ☐ Did not complete high school
5. How would you rate your general health? ☐ Excellent ☐ Good ☐ Fair ☐ Poor
6. Your household income last year was: ☐ < $20,000 ☐ $20,000-$38,000 ☐$38,001 – $60,000 ☐ $60,001-$100,000 ☐> $100,000
7. Were you born outside of the U.S./U.S. territories? ☐ Yes ☐ No

Next, I would like to ask you a few <u>T</u>rue/<u>F</u>alse questions about Alzheimer's disease.

8. One symptom that can occur with AD is believing that other people are stealing one's things. True False
9. Having high blood pressure may increase a person's risk of developing AD. True False
10. Poor nutrition can make the symptoms of AD worse. True False
11. A person with AD becomes increasingly likely to fall down as the disease gets worse. True False
12. High cholesterol may increase a person's risk of developing AD. True False

Now, I would like to ask you a couple of questions about your health

13. How often do you have headache (e.g. tension or migraine)?
    - ☐None or rare (no headache or not more than general population)
    - ☐Mild (needing some medication or producing some disability)
    - ☐Chronic
14. Do you have history of chronic pain disorders such as fibromyalgia (do not include disorders as rheumatoid arthritis or hip/knee arthritis)?
    - ☐None or rare (no pain or not more than the general population)
    - ☐Mild ((needing some medication or producing some disability)
    - ☐Chronic
15. How would you rate your memory at the present time? (circle one)

    Excellent Good Average Below average Poor

For the last part of the survey, I will ask you a few questions about the procedure lumbar puncture.

16. Do you know what a lumbar puncture (or a spinal tap) is? Yes No

17. Have you had a lumbar puncture (or a spinal tap) in the past? Yes No

18. Has anyone you know (other than yourself) had a lumbar (or spinal tap)? Yes No

A lumbar puncture is used by doctors to diagnose a number of medical conditions. The spinal fluid accumulates naturally below the spinal cord, and a very small needle is placed in your back after the area is numbed to remove some of that fluid for testing.

19. Which disease(s) do you believe can be diagnosed by a lumbar puncture?

☐Meningitis ☐Multiple sclerosis ☐ Depression

☐Stroke ☐Skin diseases ☐ Parkinson's disease

☐Alzheimer's disease ☐Diseases affecting the digestive tract including liver

20. How do you see the lumbar puncture?

☐ A standard medical procedure ☐A frightening invasive procedure ☐ Not sure

21. Would you consider a lumbar puncture if
    - ☐ Your doctor can diagnose a disease you may have at a very early stage (like how a mammogram or colonoscopy is used to detect cancer early)
    - ☐ It is necessary for your doctor to tailor a treatment for a disease you already have.
    - ☐ Your doctor can determine the likelihood of you having a disease in the future (like how a cholesterol test is used to predict heart disease)
    - ☐ To further research on a disease you have
    - ☐ To further research on a disease you do not have
    - ☐ I will not have a lumbar puncture under any circumstance

Answer on the following questions ONLY IF your answer to 17 was YES (check all that apply).

22. Why did you have a lumbar puncture? ☐Clinical diagnosis ☐Research study ☐Other ________________________

23. Where did you have it done? ☐Emergency Room ☐Hospital ☐Doctor's office ☐Dedicated research center

24. Did you have any of the following complications following the lumbar puncture?
    ☐Mild headache ☐Severe headache ☐Back pain ☐Infection ☐Bleeding ☐None of these

<u>Thank you very much for your time and the info you have provided!</u>
